# Key coral symbiont lineages (family Symbiodiniaceae) are differentially associated with stony coral tissue loss disease

**DOI:** 10.1093/ismeco/ycag228

**Published:** 2026-08-08

**Authors:** Carly E Karrick, Alex J Veglia, Sonora Meiling, Kerry Maxwell, Lindsay K Huebner, Daniel M Holstein, Laura Mydlarz, Marilyn Brandt, Amy Apprill, Erinn M Muller, Adrienne M S Correa

**Affiliations:** Department of Environmental Science, Policy, and Management, University of California, Berkeley, CA 94720, United States; BioSciences Department, Rice University, Houston, TX 77005, United States; BioSciences Department, Rice University, Houston, TX 77005, United States; Department of Biology, University of Puerto Rico, Mayagüez, PR 00680, United States; Center for Marine and Environmental Studies, University of the Virgin Islands, USVI 00802, United States; South Florida Regional Laboratory, Fish and Wildlife Research Institute, Florida Fish and Wildlife Conservation Commission, Marathon, FL 33050, United States; Fish and Wildlife Research Institute, Florida Fish and Wildlife Conservation Commission, Saint Petersburg, FL 33701, United States; School of Marine and Atmospheric Sciences, Stony Brook University, Stony Brook, NY 11794, United States; Department of Oceanography & Coastal Sciences, Louisiana State University, Baton Rouge, LA 70803, United States; Department of Biology, The University of Texas at Arlington, TX 76019, United States; Center for Marine and Environmental Studies, University of the Virgin Islands, USVI 00802, United States; Department of Marine Chemistry and Geochemistry, Woods Hole Oceanographic Institution, Woods Hole, MA 02543, United States; Mote Marine Laboratory & Aquarium, Sarasota, FL 34236, United States; Department of Environmental Science, Policy, and Management, University of California, Berkeley, CA 94720, United States; BioSciences Department, Rice University, Houston, TX 77005, United States

**Keywords:** stony coral tissue loss disease (SCTLD), Symbiodiniaceae, symbiosis, coral disease, ITS-2, microbial ecology

## Abstract

Stony coral tissue loss disease (SCTLD) has caused unprecedented coral mortality across the Caribbean, and its etiology remains unresolved. SCTLD affects >30 coral species that differ in their associations with dinoflagellate endosymbionts (family Symbiodiniaceae), and symbiont lineage-level dynamics may underlie variation in holobiont disease response. We analyzed 491 apparently healthy and SCTLD-affected corals in Florida (USA) and the United States Virgin Islands, representing 9 coral species sampled *in situ* or during an SCTLD transmission experiment. Based on the internal transcribed spacer-2 (ITS-2) region of rDNA, we identified 114 Symbiodiniaceae ITS-2 type profiles spanning 6 genera. Symbiodiniaceae composition varied significantly by coral species, disease susceptibility group, tissue health state, and region. In the corals *Orbicella annularis* and *Colpophyllia natans*, lesion-adjacent tissues had enriched relative abundance of a *Durusdinium* lineage, whereas *Symbiodinium* and *Cladocopium* lineages were relatively more abundant in apparently healthy tissues. Accounting for biogeography and host identity, our results suggest that in corals harboring multiple Symbiodiniaceae lineages, more susceptible lineages are lost first, leaving relatively resistant lineages in lesion-adjacent tissue until sloughing occurs. These findings indicate SCTLD acts as a selective filter on coral symbiont assemblages. Our results provide the first multi-species, multi-region evidence of fine-scale associations between Symbiodiniaceae lineages and SCTLD, establishing a framework for testing lineage-specific disease susceptibility in future experiments.

## Introduction

In 2014, the most pervasive and deadly coral disease to date, stony coral tissue loss disease (SCTLD), emerged off the coast of Miami, Florida, USA [1]. SCTLD has since spread throughout the Caribbean, affecting >30 coral species in >30 countries/territories [2]. SCTLD presents as focal or multi-focal areas of acute to subacute tissue loss [3] that can rapidly spread throughout an entire coral in a few weeks or months [4]. Due to its unprecedented rate of transmission and lesion progression, SCTLD has spurred rapid reef decline [5–7], causing >50% reductions in coral cover in some areas [8]. At least some of the diverse microorganisms that form symbiotic partnerships with reef-building corals (e.g. endosymbiotic dinoflagellates in the family Symbiodiniaceae, bacteria, and viruses; [9]) likely influence and/or are influenced by SCTLD dynamics, given their roles in coral holobiont health and function. For example, enrichment of certain bacterial [10–21] and viral [22–24] groups have been documented in SCTLD-affected tissues, though the disease’s etiology remains unresolved (reviewed in [25]).

Several lines of evidence suggest that Symbiodiniaceae are central to SCTLD pathology and dynamics. Meiling et al. (2020) [4] documented that SCTLD lesion progression halted in *Orbicella annularis* corals following episodes of mass loss of Symbiodiniaceae from corals (i.e. coral bleaching) based on 3D photogrammetry. Microscopy-based studies indicate that SCTLD results in the breakdown of the coral–Symbiodiniaceae symbiosis [26–28] and SCTLD-affected coral tissues contain Symbiodiniaceae cells with signs of putative viral infection ([29], but see [30, 31]). Metatranscriptomic evidence corroborates these findings, documenting increased expression of symbiont-degrading genes and/or other genes that may cause dysbiosis in SCTLD-affected corals [26, 32–34]. Further, Symbiodiniaceae-generated metabolites are linked to differences in profiles of SCTLD-affected versus apparently healthy corals [35].

A diversity of Symbiodiniaceae have been documented in SCTLD-susceptible coral species, including members of the genera *Symbiodinium*, *Breviolum*, *Cladocopium*, and *Durusdinium* (e.g. [30]); this is unsurprising given known variation in Symbiodiniaceae composition across coral species and biogeographic regions (e.g. [36–39]). Although a relatively narrow range of host species, timepoints, and sample types have been assessed, coral species with low to no SCTLD susceptibility are generally dominated by the genus *Symbiodinium*, whereas moderately susceptible coral species harbor more variable Symbiodiniaceae assemblages (including *Breviolum*, *Cladocopium*, and/or *Durusdinium*), and coral species with high susceptibility tend to be dominated by *Breviolum* spp. [8]. Dennison et al. (2021) [40] manipulated Symbiodiniaceae composition in 5 Florida coral species (2–15 genotypes per species) via experimental bleaching in 2020 and found that corals dominated by *Breviolum* symbionts had significantly reduced survival following exposure to SCTLD relative to corals dominated by *Cladocopium* or *Durusdinium*, ultimately proposing an SCTLD susceptibility hierarchy for corals dominated by distinct Symbiodiniaceae genera: (from most to least susceptible) *Breviolum* > > *Cladocopium* > *Durusdinium* > > *Symbiodinium*. Karp et al. (2023) attributed this susceptibility hierarchy to variation between Symbiodiniaceae genera (rather than hosts) by showing physiological decline in some Symbiodiniaceae cultures exposed to SCTLD-associated water, with *Breviolum minutum* exhibiting more severe decline, followed by *Cladocopium goreaui* and *Durusdinium trenchii*; *Symbiodinium microadriaticum* was not affected [41]. Another SCTLD transmission experiment in Florida documented increased risk of SCTLD in *Orbicella faveolata* (10 genotypes) and *Pseudodiploria clivosa* (3 genotypes) corals with a higher ratio of *Breviolum* to *Durusdinium* Symbiodiniaceae [42]. In contrast, other SCTLD transmission experiments conducted in 2019 and/or 2020 observed increased disease transmission [43] and severity [26] in Florida *Montastraea cavernosa* corals (*n* = 41) and US Virgin Islands *Colpophyllia natans* corals (*n* = 8) dominated by *Durusdinium*, relative to conspecifics dominated by *Cladocopium*. Additionally, the presence of *Durusdinium* was associated with SCTLD in 90 *O. faveolata* on reefs in Florida in 2019 [44].

Associations between Symbiodiniaceae diversity and SCTLD incidence have yet to be tested at fine-scale taxonomic resolution (e.g. Symbiodiniaceae species, lineages, and populations). This is an important next step given that the genus level is recognized as insufficient for characterizing Symbiodiniaceae physiology and ecology [45]. For example, *B. minutum*, but not *Breviolum psygmophilium*, exhibited negative impacts from water exposed to SCTLD-affected corals in a culture-based experiment [41]. We therefore comprehensively investigated how the relative and differential abundance of Symbiodiniaceae lineages varies across coral susceptibility groups and tissue health status. Our results provide the first evidence for Symbiodiniaceae internal transcribed spacer-2 (ITS-2) type profiles associated with SCTLD lesions and highlight nuances in these associations across coral species and regions.

## Materials and methods

### Sample collection

**Figure 1:**
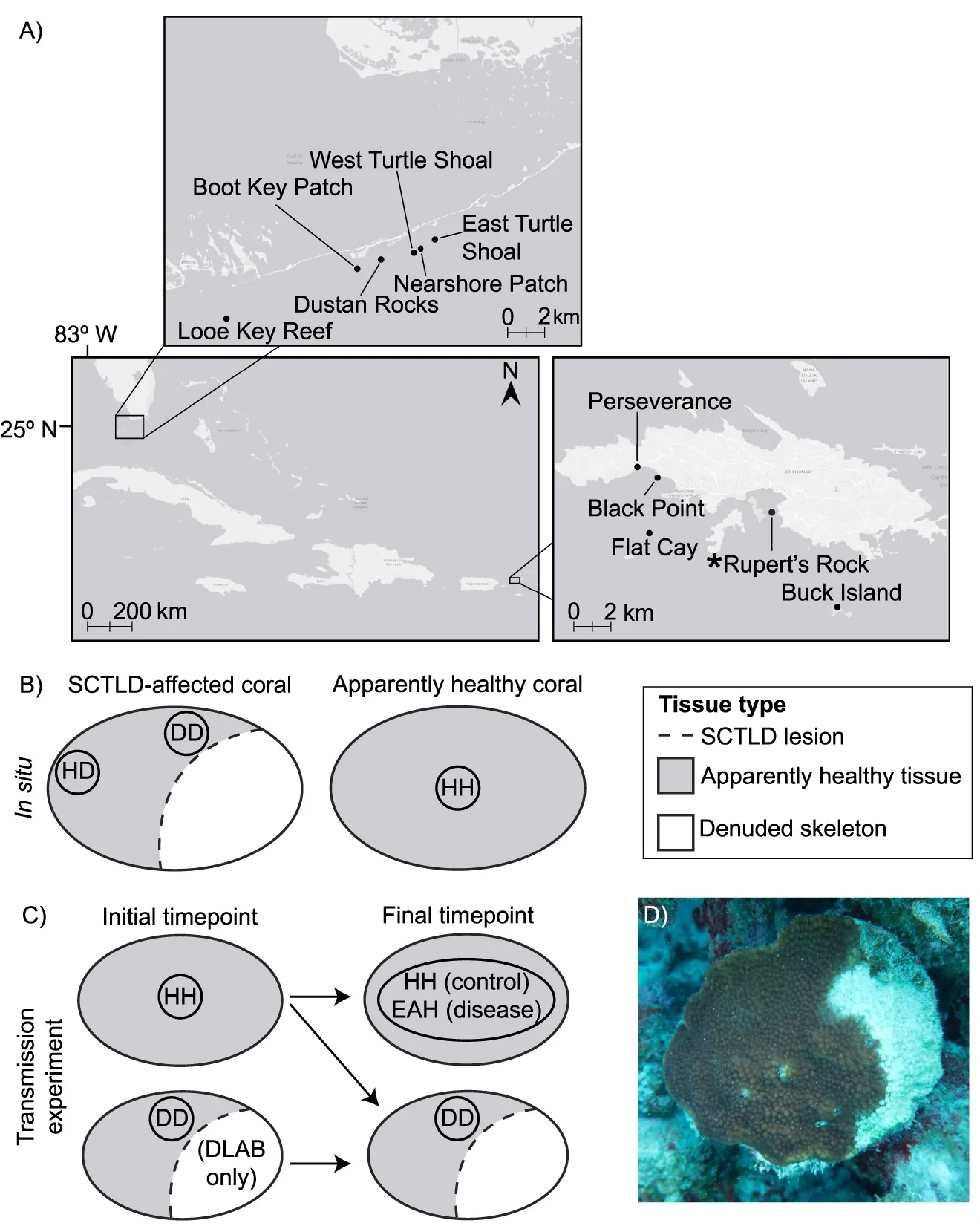
(A) Between 2018 and 2020, Caribbean stony corals were collected for Symbiodiniaceae diversity analyses from six sites in the Florida Keys, USA (A, top) and five sites in the US Virgin Islands (A, bottom right). The asterisk (Rupert’s Rock) indicates the site where corals included in the SCTLD transmission experiment were originally collected. (B) For corals sampled *in situ*, two samples (HD, DD) were collected from SCTLD-affected corals, and then one sample (HH) was collected from a nearby apparently healthy conspecific coral. (C) For corals sampled during the SCTLD transmission experiment, initial fragments were all HH, except diseased *D. labyrinthiformis* corals used as central disease transmitters in the disease treatment. Final fragments were HH if the fragment was in the control treatment (i.e. not exposed to a central SCTLD-affected coral), exposed apparently healthy (EAH) if the fragment did not develop visible signs of SCTLD but was exposed to a central diseased coral, or DD if the fragment developed an SCTLD lesion. (D) An example of an active SCTLD lesion on an *O. faveolata* coral. HH: apparently healthy tissue on an apparently healthy coral; HD: apparently healthy tissue from a SCTLD-affected coral; DD: SCTLD lesion-adjacent tissue; EAH: apparently healthy tissue on corals exposed to a central SCTLD-affected coral in the transmission experiment.

We examined a total of 491 coral tissues/fragments collected between 2018 and 2020 from reefs in Florida (USA) and the US Virgin Islands (USVI, Fig. 1A, Table 1). From Florida, we examined 215 samples collected *in situ* from 6 epidemic sites in 2018 [13, 18]. From the USVI, we examined 110 samples collected *in situ* from 4 reef sites in 2019–2020. Samples from nine coral species were included, representing a range of reported susceptibilities to SCTLD (Table 1). These susceptibility categories reflect the Florida Department of Environmental Protection’s SCTLD case definition (https://floridadep.gov/sites/default/files/Copy%20of%20StonyCoralTissueLossDisease_CaseDefinition%20final%2010022018.pdf); however, regional variation has been reported [28, 31, 46] but see [47]. Of the total samples, 325 were sampled *in situ* and were attempted to be sampled as sets of SCTLD-affected and apparently healthy conspecifics (Fig. 1B):

- Two samples were collected from each diseased coral: one from tissue adjacent to the gross tissue loss lesion (DD) and one from apparently healthy tissue on the same coral (HD).
- One sample was then collected from a nearby conspecific coral that was apparently healthy (HH).

**Table 1:** Stony coral samples collected and analyzed for Symbiodiniaceae diversity.

**SCTLD susceptibility**	**Coral species**	**Region**	**Year**	**Sample origin (experiment/field)**	**Health state**	***n* (total)**	***n* (copy # corrected analysis excluding initial experimental samples and samples with *Gerakladium*)**
Low	*Porites astreoides*	USVI	2019	Experiment	HH	16	7
					EAH	2	2
					DD	6	6
				Field	HH	13	12
					HD	0	0
					DD	0	0
Moderate	*Montastraea cavernosa*	Florida	2018	Field	HH	16	8
					HD	24	15
					DD	25	14
		USVI	2019	Experiment	HH	16	8
					EAH	5	5
					DD	3	3
			2020	Field	HH	5	5
					HD	6	6
					DD	4	4
Moderate	*Orbicella annularis*	USVI	2019	Experiment	HH	13	6
					EAH	1	1
					DD	5	5
				Field	HH	11	11
					HD	10	9
					DD	11	11
Moderate	*Orbicella faveolata*	Florida	2018	Field	HH	1	1
					HD	2	2
					DD	2	2
Moderate	*Siderastrea siderea*	Florida	2018	Field	HH	0	0
					HD	1	1
					DD	1	1
		USVI	2019	Experiment	HH	16	8
					EAH	1	1
					DD	7	7
High	*Colpophyllia natans*	Florida	2018	Field	HH	16	14
					HD	25	23
					DD	25	22
		USVI	2019	Experiment	HH	16	7
					EAH	0	0
					DD	8	7
				Field	HH	10	10
					HD	10	10
					DD	10	10
High	*Diploria labyrinthiformis*	USVI	2019	Experiment	HH	15	7
					EAH	0	0
					DD	12	4
High	*Meandrina meandrites*	Florida	2018	Field	HH	3	2
					HD	5	3
					DD	5	4
		USVI	2020	Field	HH	1	1
					HD	3	3
					DD	4	4
High	*Pseudodiploria strigosa*	Florida	2018	Field	HH	16	14
					HD	24	23
					DD	24	18
		USVI	2019	Experiment	HH	16	8
					EAH	2	1
					DD	6	6
				Field	HH	4	4
					HD	4	4
					DD	4	4

Table 1: Stony coral samples collected and analyzed for Symbiodiniaceae diversity (internal transcribed spacer-2 region of rDNA) from the USVI and Florida (USA), based on SCTLD susceptibility (National Oceanic and Atmospheric Association SCTLD Case Definition: https://floridadep.gov/sites/default/files/Copy%20of%20StonyCoralTissueLossDisease_CaseDefinition%20final%2010022018.pdf), reef region, year of collection, sample type, and health state. HH: apparently healthy tissue collected from an apparently healthy coral; HD: apparently healthy tissue collected from a SCTLD-affected coral; DD: SCTLD lesion-adjacent tissue; EAH: apparently healthy tissue from an apparently healthy coral exposed to a diseased coral in the transmission experiment.

Per site in Florida, up to five diseased corals and up to three apparently healthy corals were sampled. In the USVI, diseased and healthy samples were collected as matched sets when possible. However, circumstances prevented this in some cases; for example, only apparently healthy *Porites astreoides* could be sampled *in situ*. The remainder of the samples in this study (*n* = 166 samples representing 64 coral genotypes) were taken during a wet lab-based SCTLD transmission experiment [28].

One week prior to the start of the experiment, eight apparently healthy corals representing eight genotypes of each of six coral species (*C. natans*, *M. cavernosa*, *O. annularis*, *P. astreoides*, *Pseudodiploria strigosa*, and *Siderastrea siderea*) were collected from Rupert’s Rock in the USVI and fragmented in half. After acclimation, one fragment per genet was transferred to each of a control and disease treatment bin. Each treatment was comprised of eight plastic bins, and each bin contained a single *Diploria labyrinthiformis* coral (collected one day prior to the start of the experiment) arranged in the center surrounded by six equidistant coral fragments, one per species (see Fig. 1 in [28] for example images of a control and a treatment bin). In the control treatment, the central coral was an apparently healthy *D. labyrinthiformis* collected from Rupert’s Rock, while that of the disease treatment was an SCTLD-affected *D. labyrinthiformis* collected from Flat Cay. During this experiment, one sample was taken from each genet at the start (*n* = 63) and one from each fragment at the end of the trial (*n* = 103) when possible (either at least 12 hours after the appearance of an expanding SCTLD lesion or after 8 days; Fig. 1C). All final samples from the control treatment were labeled HH since they did not develop visible signs of SCTLD. Final samples exposed to the SCTLD-affected coral were categorized as DD if the fragment developed disease signs (see example lesion in Fig. 1D) and EAH (exposed apparently healthy) if no disease signs were visible. The lesion progression rate (cm^2^/day) was calculated for each experimental DD fragment by using ImageJ [48] to measure lesion growth in photos taken at the initial observation of a lesion and before processing.

### DNA extraction and sequencing

Coral tissues collected in Florida were flash frozen and stored at −80°C as described by Clark et al. (2021) [13]. Samples collected in the USVI were preserved in DNA/RNA Shield (Zymo Research) and vortexed at maximum speed for 20 minutes to ensure sufficient cell lysis before storage at −80°C. DNA was extracted from each sample using the ZymoBIOMICS DNA/RNA Miniprep Kit (Zymo Research) according to the manufacturer’s instructions. The ITS-2 region of Symbiodiniaceae rDNA was amplified using the SYM_Var primer pair [49]: SYM_VAR_5.8SII (5′-GAATTGCAGAACTCCGTGAACC-3′) and SYM_VAR_REV (5′-CGGGTTCWCTTGTYTGACTTCATGC-3′). PCR reagents included 2 μL of DNA, 0.42 μL of forward and reverse primers, 15 μL of DNase/RNase-free water, and 17.5 μL of EconoTaq PLUS GREEN 2× Master Mix. After 10 minutes at 74°C, PCR conditions included 32 cycles of 94°C for 1 minute, 59°C for 1 minute, and 74°C for 2 minutes, followed by 74°C for 7 minutes. Sequencing was performed on an Illumina MiSeq PE300 platform at Oregon State University’s Center for Quantitative Life Sciences (OSU CQLS, Corvallis, OR, USA), and raw sequencing data are available in NCBI BioProjects PRJNA955224, PRJNA1230223, and PRJNA1375785.

### Bioinformatic processing and statistical analyses

Untrimmed, demultiplexed sequence libraries were imported to the online SymPortal framework (symportal.org; [50]) for taxonomic identification of ITS-2 type profiles using the latest remote database (as of February 2025). SymPortal partitions Symbiodiniaceae lineages into distinct genotypes called ITS-2 type profiles, where each defining intragenomic variant is separated by a dash, listed in order of decreasing abundance. The “profiles.absolute.abund_and_meta” file from the SymPortal output was used for downstream analyses; this file contains the absolute abundance of each ITS-2 type profile per sample.

For statistical analyses and visualizations of our data, the counts table generated by SymPortal was imported into RStudio (version 2023.06.2 + 561; [51]) using the package phyloseq (v.1.48.0; [52]). To import these data as a phyloseq object, an Excel file was created containing separate sheets with (i) the counts table transposed from the SymPortal output (i.e. ITS-2 type profile as rows and samples as columns), (ii) the taxonomy table with ITS-2 type profiles as rows and taxonomy information as columns, and (iii) the sample table with each sample ID (from the counts table columns) as rows and relevant metadata as columns (File S1). To wrangle these data, several packages included in tidyverse (v2.0.0; [53]) were used. Samples were filtered in various steps described below and summarized in Fig. S1. A rarefaction curve was generated with the rarecurve() function of vegan (v2.7-2; [54]) to determine adequate sequencing depth for downstream analyses (Fig. S2). Based on this curve, samples with <1000 reads (*n* = 49) were removed, whereas 442 samples were retained for downstream analysis. The 49 samples with <1000 reads were comprised of 15 HH, 14 HD, and 20 DD samples; 46 of these samples were from Florida and 3 were from the USVI. Of the 442 samples retained, 61 samples (all initial samples from the transmission experiment) were excluded from statistical analyses to avoid repeated measures between initial and final HH samples of the control treatment. Wilcoxon tests (wilcox.test() function of R package stats v4.4.1; [51]) and Kruskal–Wallis tests (kruskal.test() function of R package stats v4.4.1; [51]) were used to assess whether sequencing depth varied among biogeographic regions, sample origins (experiment/field), health states, and host species since these data violated the parametric assumption of normality (Shapiro–Wilk test W = 0.88811, *P* = 4.716 × 10^−16^). If overall statistical significance was observed, pairwise comparisons were performed with Dunn’s tests with a Benjamini–Hochberg correction using the dunn_test() function of the R package rstatix (v0.7.3; [55]), and differences were summarized with compact letter displays with the cldList() function of the R package rcompanion (v2.5.2; [56]). Effect sizes were computed for these pairwise comparisons by dividing the test statistic by the square root of the total number of samples (*n* = 381).

Because variation in ITS-2 copy number among Symbiodiniaceae genera can bias composition estimates towards lineages with higher copy numbers [45], correction for variation in copy number was conducted by dividing absolute counts of each ITS-2 type profile by the average copy number of its associated genus reported by Saad et al. (2020) [57], as performed by Howe-Kerr et al. (2023) [30]. Because Saad et al. (2020) [57] did not report a copy number estimate for *Gerakladium*, samples containing this genus (*n* = 7) were omitted from the copy-number-corrected analyses. The 374 samples analyzed with copy number correction are summarized in the last column of Table 1. After correcting for copy number variation, data were normalized by converting from absolute to relative abundance per sample by dividing the absolute counts of each ITS-2 type profile by the total number of absolute counts of all ITS-2 type profiles in each sample.

The relative abundances of Symbiodiniaceae genera and ITS-2 type profiles for all samples were plotted using ggplot2 (v.3.5.1; [58]). PERMANOVA tests of Bray–Curtis dissimilarity matrices were used with the adonis() function within the vegan package (v2.6.10; [54]) to test for significant differences in Symbiodiniaceae ITS-2 type profile diversity between tissue health states and SCTLD susceptibility groups in the entire dataset. Pairwise tests were conducted with pairwiseAdonis (v0.4.1; [59]) with a Bonferroni correction. Additional PERMANOVA tests were run per coral species to assess biogeographic variation in ITS-2 type profile composition between Florida and the USVI. For coral species that varied biogeographically, region-specific PERMANOVAs were used to compare ITS-2 type profile diversity between tissue health states. If no significant biogeographic variation was detected, comparisons between tissue health states within coral species included both Florida and the USVI. Heterogeneous dispersion was observed between susceptibility groups, *C. natans* regions, *P. strigosa* regions, and *P. strigosa* health states in the USVI; so, 34, 52, 34, and 10 samples, respectively, were randomly subsampled before testing for differences between groups. Due to insufficient sample size of one or more tissue types, differences in Symbiodiniaceae ITS-2 type profiles could not be assessed across all health states (HH, HD, and DD) in *P. astreoides* (PERMANOVA conducted on HH-DD only), *O. faveolata* (no PERMANOVAs conducted), *S. siderea* (PERMANOVA conducted on HH-DD only), *D. labyrinthiformis* (PERMANOVA conducted on HH-DD only), and *Meandrina meandrites* (PERMANOVA conducted on HD-DD only).

To assess the specificity of associations of Symbiodiniaceae ITS-2 type profiles with coral species, biogeographic regions, and SCTLD susceptibility groups, ggplot2 [58] was used to create bar plots of the proportion of samples containing each Symbiodiniaceae lineage in each coral species, region, or susceptibility group. The phylogenetic relatedness of these ITS-2 type profiles was visualized with dendrograms depicting hierarchical clustering of UniFrac-derived between-ITS-2-type-profile distances based on square-root-transformed abundances of defining intragenomic variants provided with the SymPortal output. Dendrograms were created with the ggdendro R package (v0.2.0; [60]) and hierarchical clusters with the hclust() function of the stats package (v4.2.1; [51]).

To test whether certain ITS-2 type profiles were differentially relatively abundant between health states within a coral species, Analysis of Composition of Microbiomes with Bias Correction (ANCOM-BC) with Bonferroni correction was used. For the ANCOM-BC analyses, the R package ANCOMBC (v.2.6.0; [61]) was used with default parameters unless otherwise specified. ANCOM-BC was conducted on subsets of the data per coral species with the ancombc2() function (tax_level = “Species,” formula = “health,” group = “health,” p.adj.method = “bonferroni,” prv_cut = 0.01). To test each possible pairwise comparison except those with EAH since *n* < 10 per coral species, ANCOM-BC was conducted with either HH or HD as a baseline. This analysis could not be performed on *O. faveolata* due to low sample size per health state. For each ANCOM-BC analysis, the log fold change ± standard error for ITS-2 type profiles that were relatively differentially abundant with statistical significance (p_adj < 0.05) was plotted using ggplot2 (v.3.5.1; [58]).

For samples from the SCTLD transmission experiment, the percentage of coral genotypes with altered abundance of Symbiodiniaceae lineages with significant differential relative abundance (based on the ANCOM-BC analysis) from the initial to final timepoint of the experiment was calculated per coral species. These percentages could not be statistically tested due to low sample sizes per coral species. Furthermore, ANCOM-BC was not run on the subset of experimental genotypes containing multiple ITS-2 type profiles in the initial timepoint (*n* = 11) due to low sample size per coral species. The mean lesion progression rate was compared across samples dominated by distinct ITS-2 type profiles using Kruskal–Wallis tests. Kruskal–Wallis tests were performed with the kruskal.wallis function in the package stats (v4.4.1; [51]) for all coral species in the SCTLD transmission experiment except *O. annularis* (all experimental samples of this species were dominated by the same ITS-2 type profile).

## Results

Ranging from 1155 to 240 682, an average of 58 615 reads were recovered per sample from the 442 samples (excluding the 49 samples with <1000 total reads). When the 61 samples taken at the start of the SCTLD transmission experiment were also excluded, an average of 58 593 reads were recovered per sample (*n* = 381). In these 381 samples, sequencing depth did not significantly differ across biogeographic regions (Wilcoxon rank sum test W = 18 314, *P* = .7087), sample origins (experiment/field; Wilcoxon rank sum test W = 13 754, *P* = .6177), or health states (Kruskal–Wallis rank sum test χ^2^ = 0.10234, df = 3, *P* = .9916). Sequencing depth differed across host species (Kruskal–Wallis rank sum test χ^2^ = 56.719, df = 8, *P* = 2.043 × 10^−9^), with effect sizes ranging from small to moderate magnitudes (|r| ≈ 0.008–0.276). *D. labyrinthiformis* had the lowest median sequencing depth, followed by *P. astreoides*, *P. strigosa*, *M. meandrites*, *C. natans*, *O. annularis*, *S. siderea*, *M. cavernosa*, and *O. faveolata* (Fig. S3). *O. faveolata* was the only coral species with significantly different sequencing depth in all pairwise comparisons with other host species (Fig. S3). Despite significant variation in sequencing depth between some coral species, many pairwise comparisons (particularly among *O. annularis*, *S. siderea*, *C. natans*, *M. meandrites*, and *P. strigosa*) were not statistically significant and had low effect sizes (|r| < 0.1) (Fig. S3).

**Figure 2:**
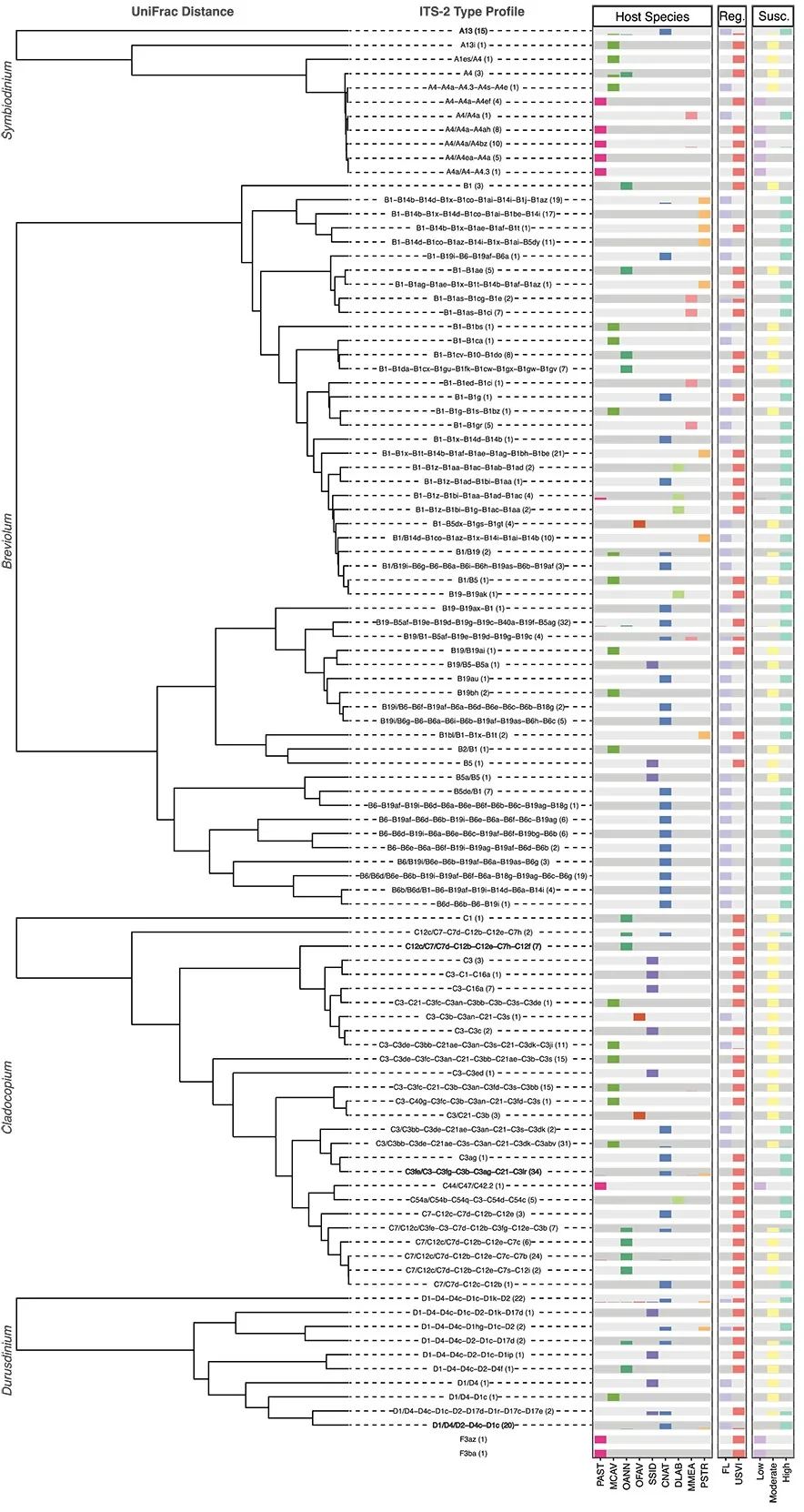
Specificity of Symbiodiniaceae ITS-2 type profiles to host species, biogeographic regions (“Reg.”), or SCTLD susceptibility groups (“Susc.”). One Symbiodiniaceae lineage is presented per row, with its name followed by the number of samples in which it was detected (in parentheses). The relatedness of ITS-2 type profiles is presented with a dendrogram of hierarchical clustering of UniFrac between-profile distances. ITS-2 type profiles are arranged by genus; from top to bottom: *Symbiodinium*, *Breviolum*, *Cladocopium*, and *Fugacium*. To the right of each Symbiodiniaceae lineage, bars represent the proportion of all the samples containing that ITS-2 type profile for each host species, biogeographic region, or SCTLD susceptibility group. **Bolded ITS-2 type profiles** are differentially abundant across health states based on ANCOM-BC analyses. PAST: *Porites astreoides*; MCAV: *Montastraea cavernosa*; OANN: *Orbicella annularis*; OFAV: *O. faveolata*; SSID: *Siderastrea siderea*; CNAT: *Colpophyllia natans*; DLAB: *Diploria labyrinthiformis*; MMEA: *Meandrina meandrites*; PSTR: *Pseudodiploria strigosa*; FL: Florida, USA; USVI: US Virgin Islands.

From the reads of these 381 samples, SymPortal identified 108 ITS-2 type profiles (see Table S1 for ITS-2 type profiles present in all 442 samples and File S1 for the SymPortal output). These 108 type profiles spanned 6 Symbiodiniaceae genera: 11 type profiles of *Symbiodinium*, 52 of *Breviolum*, 28 of *Cladocopium*, 10 of *Durusdinium*, 2 of *Fugacium*, and 5 of *Gerakladium* (Table S1). *Fugacium* and *Gerakladium* were detected in few samples (*n* = 1 and *n* = 7, respectively) and were generally present at low relative abundances (<0.2% and <22%, respectively) of the total Symbiodiniaceae reads recovered from a sample. No patterns in the distribution of *Gerakladium* were apparent for host species, geographic location, susceptibility category, or health state. A single ITS-2 type profile was identified from most samples (Figs S4–S12); if multiple ITS-2 type profiles were detected in a sample, there was generally only one type profile per Symbiodiniaceae genus (but see OANN_13, OANN_14, OANN_20, CNAT_8, CNAT_16, and CNAT_41; Figs S6 and S9). All subsequent statistical results are based on samples (*n* = 374) that could be corrected for copy number variation (i.e. did not contain *Gerakladium*) and represented final (or only) sampling time points (i.e. initial experimental samples were excluded). After removing these samples, 102 ITS-2 type profiles remained: 11 of *Symbiodinium*, 52 of *Breviolum*, 27 of *Cladocopium*, 10 of *Durusdinium*, and 2 of *Fugacium* (Fig. 2).

### Coral species vary biogeographically in the Symbiodiniaceae ITS-2 type profiles they harbor

Symbiodiniaceae composition varied across coral species and between biogeographic regions (Fig. 2). Of the nine coral species in this study, five were sampled in both Florida and the USVI, and four of these coral species (*M. cavernosa*, *C. natans*, *M. meandrites*, and *P. strigosa*) had sufficient sample sizes to test for significant differences in Symbiodiniaceae ITS-2 lineage diversity between regions. These four species had significantly different Symbiodiniaceae ITS-2 type profile compositions between regions (PERMANOVA *P* < .05; Figs S5, S9, S11, and  S12, and Table S2). Most ITS-2 type profiles (~90%; 92 of 102) were specific to one region, with ~82% (9 of 11) of *Symbiodinium* lineages, ~96% (50 of 52) of *Breviolum* lineages, ~89% (24 of 27) of *Cladocopium* lineages, 70% (7 of 10) of *Durusdinium* lineages, and 100% (2 of 2) of *Fugacium* lineages only being detected in either Florida or the USVI (Fig. 2).

### Symbiodiniaceae ITS-2 type profile relative abundance varies with coral susceptibility to SCTLD and tissue health state

Most Symbiodiniaceae lineages were specific to coral species (~81%; 83 of 102) and/or susceptibility groups (~85%; 87 of 102) (Fig. 2). Coral species with differential susceptibility to SCTLD had significantly different Symbiodiniaceae ITS-2 type profile compositions (PERMANOVA df = 2, SumOfSqs = 1.6519, R^2^ = 0.2475, F = 1.9735, *P* = .008; Table S2). The coral species with low susceptibility to SCTLD (*P. astreoides*) was dominated by *Symbiodinium* ITS-2 type profiles, whereas moderately susceptible corals (*M. cavernosa, O. annularis, O. faveolata,* and *S. siderea*) were dominated by *Cladocopium* ITS-2 type profiles and contained *Breviolum* lineages. Highly susceptible corals (*C. natans*, *D. labyrinthiformis*, *M. meandrites*, and *P. strigosa*) were dominated by *Breviolum* type profiles. *Durusdinium* lineages were detected in ~14% of corals analyzed (53 of 374), including ~3.7% (1 of 27) of *P. astreoides* samples, ~2.9% (2 of 68) of *M. cavernosa*, ~9.3% (4 of 43) of *O. annularis*, 40% (2 of 5) of *O. faveolata*, ~33% (6 of 18) of *S. siderea*, ~28% (29 of 103) of *C. natans*, ~18% (2 of 11) *D. labyrinthiformis*, 0% (0 of 17) *M. meandrites*, and ~11% (9 of 82) of *P. strigosa*. Fifteen (~28%) of these 53 *Durusdinium*-containing corals were dominated by *Durusdinium* lineages; coral species with *Durusdinium*-dominated samples included *O. annularis*, *O. faveolata*, *S. siderea*, *C. natans*, and *P. strigosa*.

**Figure 3:**
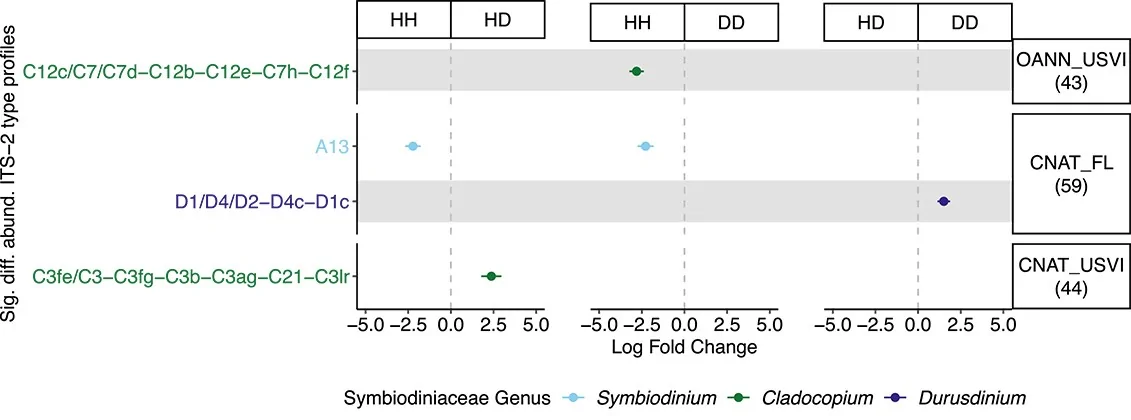
Log-fold change ± standard error of Symbiodiniaceae ITS-2 rDNA type profiles present at significantly higher relative abundance (p_adj > 0.05) in pairwise comparisons of different tissue health states within the corals *O. annularis* (OANN) and *C. natans* (CNAT) from Florida, USA (FL), and the US Virgin Islands (USVI). Within each boxed coral species, dots are located to the left or right of the dashed line at 0 log-fold change based on the health state they were significantly more relatively abundant in per pairwise comparison designated by the pair of tissue health states identified in the top panel. HH: apparently healthy tissue on an apparently healthy coral; HD: apparently healthy tissue on an SCTLD-affected coral; DD: SCTLD lesion-adjacent tissue. Sample sizes are shown under each coral species/region. No significant differences in relative abundance were detected for Symbiodiniaceae ITS-2 type profiles in *M. cavernosa* or *P. strigosa*. *P. astreoides*, *O. faveolata*, *S. siderea*, *D. labyrinthiformis*, and *M. meandrites* had too small of sample sizes (*n* < 10 each) for ANCOM-BC analysis, so ITS-2 type profiles for these corals are not included.

Across all coral species, tissue health states did not contain distinct Symbiodiniaceae ITS-2 type profile diversity (PERMANOVA *P* = .506; Table S2). After accounting for regional variation, Symbiodiniaceae composition was not significantly different between tissue health states in any individual coral species (see PERMANOVA results in Table S2). Based on overall ANCOM-BC results, ~3.9% (4 of 102) of ITS-2 type profiles had differential relative abundance across tissue health states, with 0%–8.7% of ITS-2 type profiles relatively differentially abundant within individual coral species (Table 2). Overall, a *Durusdinium* lineage was significantly enriched in DD tissue, whereas a *Symbiodinium* and a *Cladocopium* lineage were relatively more abundant in HH tissue, and a different *Cladocopium* lineage was relatively more abundant in HD tissue (Fig. 3). Specifically, *Durusdinium* D1/D4/D2-D4c-D1c was relatively more abundant in Florida *C. natans* (high SCTLD susceptibility) DD tissue, whereas *Symbiodinium* A13 was relatively less abundant in these tissues. *Cladocopium* C3fe/C3-C3fg-C3b-C3ag-C21-C3lr was relatively more abundant in HD compared to HH tissues of USVI *C. natans*, and *Cladocopium* C12c/C7/C7d-C12b-C12e-C7h-C12f was relatively more abundant in *O. annularis* (only sampled in the USVI; moderate SCTLD susceptibility) HH tissue than DD tissue (Fig. 3).

**Table 2:** Number of ITS-2 type profiles per coral species that exhibit significant differential relative abundance

**Coral species**	**# (%) relatively differentially abundant ITS-2 type profiles**	**Total # ITS-2 type profiles**
*Porites astreoides*	0 (0%)	13
*Montastraea cavernosa* (FL)	0 (0%)	11
*M. cavernosa* (USVI)	0 (0%)	12
*Orbicella annularis*	1 (5.6%)	18
*Orbicella faveolata*	0 (0%)	4
*Siderastrea siderea*	0 (0%)	13
*Colpophyllia natans* (FL)	2 (8.7%)	23
*C. natans* (USVI)	1 (6.3%)	16
*Diploria labyrinthiformis*	0 (0%)	5
*Meandrina meandrites* (FL)	0 (0%)	7
*M. meandrites* (USVI)	0 (0%)	3
*Pseudodiploria strigosa* (FL)	0 (0%)	7
*P. strigosa* (USVI)	0 (0%)	7
**Total**	**4 (3.9%)**	**102**

Table 2: ANCOM-BC results of the number of ITS-2 type profiles with significant differential relative abundance across tissue health states and the total number of observed ITS-2 type profiles in each coral species. *M. cavernosa*, *C. natans*, *M. meandrites*, and *P. strigosa* are split into regional datasets due to significantly different Symbiodiniaceae ITS-2 diversity in Florida, USA (FL) and the USVI. The final, bolded row includes all coral species grouped together.

Differentially abundant ITS-2 type profiles identified by the ANCOM-BC analysis exhibited within-group heterogeneity across coral-species-specific regional subsets. For example, ~23.7% (14 of 59) of Florida *C. natans* samples contain the *Durusdinium* type profile that was enriched in DD tissue (Table 3). Similar examples exist for USVI *C. natans* and *O. annularis* (Table 3). The Symbiodiniaceae lineages with differential relative abundance between health states in *O. annularis* and *C. natans* were specific to coral species, and the lineages relatively differentially abundant in *C. natans* were specific to regions. Overall, Symbiodiniaceae ITS-2 type profile relative abundance did not vary between tissue health states in *M. cavernosa* or *P. strigosa*.

**Table 3:** Number of samples containing ITS-2 type profiles exhibiting significant differential relative abundance.

**Coral species**	**ITS-2 type profile**	**# (%) of samples containing the ITS-2 type profile**	**Total # of samples per coral species**
*Orbicella annularis*	C12c/C7/C7d-C12b-C12e-C7h-C12f	7 (16.3%)	43
*Colpophyllia natans* (FL)	A13	12 (20.3%)	59
	D1/D4/D2-D4c-D1c	14 (23.7%)	
*C. natans* (USVI)	C3fe/C3-C3fg-C3b-C3ag-C21-C3lr	21 (47.7%)	44

Table 3: For each coral species with ITS-2 type profiles with significant differential relative abundance between tissue health states based on ANCOM-BC analyses, the number of samples containing the ITS-2 type profile with significant differential relative abundance and the total number of samples are provided. FL: Florida, USA; USVI: US Virgin Islands.

### Few samples contained sufficient ITS-2 type profile diversity to evaluate shifts in Symbiodiniaceae composition after experimental exposure to SCTLD

About half of the experimental coral genotypes [~56% (36 of 64)] had samples with ≥1000 reads that did not contain *Gerakladium* and represented both initial and final timepoints of a fragment that showed visible SCTLD signs. Of these genotypes, ~67% (24 of 36) contained a single ITS-2 type profile in the initial timepoint, including 1 *O. annularis*, 1 *C. natans*, and 3 *P. strigosa* genotypes. Of the 11 genotypes that contained multiple ITS-2 type profiles in the initial timepoint, only one (OANN_29) contained a Symbiodiniaceae ITS-2 type profile identified as having differential relative abundance across SCTLD health states above. In this genotype, the relative abundance of *Durusdinium* D1/D4/D2-D4c-D1c increased from the initial to the final experimental timepoint (Fig. S6). Mean lesion progression rate did not significantly differ between the dominant ITS-2 type profile of coral conspecifics in the SCTLD transmission experiment based on Kruskal–Wallis tests (Table S3).

## Discussion

This study shows that Symbiodiniaceae lineages exhibit differential association with SCTLD-affected tissues in Florida and USVI *C. natans* and USVI *O. annularis*. Based on ANCOM-BC analyses of differential abundance of Symbiodiniaceae ITS-2 type profiles, lesion-adjacent tissues had a higher relative abundance of a *Durusdinium* lineage, whereas *Symbiodinium* and *Cladocopium* lineages were relatively more abundant in apparently healthy tissues. We interpret that when these corals harbor a diversity of Symbiodiniaceae, some symbiont lineages are lost first (via pathogen-induced mortality or host-mediated expulsion), leaving relatively resistant lineages that persist until tissue sloughs as the lesion advances. Future studies can quantify the relative susceptibility of these Symbiodiniaceae ITS-2 type profiles through culture-based experiments and factorial inoculation of corals.

### Symbiodiniaceae lineage-specific associations with SCTLD in flexible coral holobionts

*Symbiodinium*, *Cladocopium*, and *Breviolum* were the most dominant Symbiodiniaceae genera in coral species with low, moderate, and high SCTLD susceptibility, respectively. *Durusdinium* lineages were detected in most coral species but typically did not dominate corals. These patterns corroborate previously proposed associations [8] and experimental observations [40]. *O. annularis* (only sampled in the USVI) and regional subsets of *C. natans* exhibited ITS-2 type profiles with significant differential relative abundance in different health states. No Symbiodiniaceae ITS-2 type profiles had differential relative abundance across multiple coral species and regions, which is unsurprising given variation in Symbiodiniaceae composition between coral species [36] and biogeographic regions [37].

Associations between Symbiodiniaceae lineages and tissue health states suggest variation in SCTLD susceptibility among symbiont lineages, which could explain differential SCTLD susceptibility among coral conspecifics [62] (although this is not tested here, it could be in a future study). Assuming the HD samples are generally representative of the coral prior to SCTLD onset, we interpret the differences detected in this study as evidence that the *Durusdinium* D1/D4/D2-D4c-D1c lineage enriched in DD tissue (relative to HD tissue) in Florida *C. natans* is relatively less susceptible to SCTLD. Similarly, we interpret that the *Cladocopium* C3fe/C3-C3fg-C3b-C3ag-C21-C3lr lineage that is relatively more abundant in HD tissue (compared to HH tissue) in USVI *C. natans* is more susceptible to SCTLD. While we did not detect differential relative abundance of any Symbiodiniaceae ITS-2 type profiles across health states in *M. cavernosa* and *O. faveolata*, previous studies have associated *Durusdinium* with increased SCTLD susceptibility in *M. cavernosa* [43] and *O. faveolata* [44] and increased SCTLD severity in *C. natans* [26]. However, it should be noted that these studies did not report Symbiodiniaceae taxonomy beyond the genus level, many apparently healthy (HH) conspecifics contained *Durusdinium* at low relative abundance, and various SCTLD-affected corals surveyed in these works did not contain *Durusdinium*. Furthermore, Titus et al. (2022) [43] and Klein et al. (2024) [44] did not compare DD and HD tissues within SCTLD-affected corals, so it cannot be assessed whether enrichment of *Durusdinium* in affected corals may have resulted from the loss of other (putatively more susceptible) lineages from lesion-adjacent tissues or if *Durusdinium* dominance was causal of an SCLTD-affected phenotype.

Coral species vary in their specificity and fidelity to Symbiodiniaceae lineages [63–65]. In high-specificity coral species dominated by a single symbiont lineage across individuals, we expect few to no differences in Symbiodiniaceae composition between diseased and healthy holobionts. In contrast, more flexible coral species may host different dominant Symbiodiniaceae lineages among holobionts or multiple lineages within a holobiont. In these cases, corals dominated by more susceptible Symbiodiniaceae lineages may tend to become SCTLD-affected. In contrast, SCTLD-resistant Symbiodiniaceae lineages may tend to remain in apparently healthy corals or persist in lesion-adjacent tissue following initial selective loss of susceptible symbionts. Such patterns are consistent with *Orbicella* spp. and *C. natans*, which often harbor variable or mixed Symbiodiniaceae assemblages [66–69] and show bleached margins around lesions (*Orbicella* only) [8]. In this study, apparently healthy *O. annularis* corals exhibited higher relative abundances of one Symbiodiniaceae ITS-2 type profile relative to SCTLD-affected individuals. Similarly, Florida and USVI *C. natans* combined exhibited three ITS-2 type profiles with significant differential relative abundance between tissue health states. “Shuffling” of Symbiodiniaceae composition has been proposed as an adaptive mechanism to environmental stressors [36, 70–72]. The rapid lesion progression rates of SCTLD (e.g. up to 7.03 cm^2^/day; [28]) relative to the slower generation times of Symbiodiniaceae [73] suggest that the differences in Symbiodiniaceae composition documented here likely result from loss of susceptible Symbiodiniaceae cells (potentially through either mortality or expulsion), rather than “shuffling” of lineages [25].

These findings are based on samples collected during epidemic outbreaks of SCTLD. The epidemic phase of a disease is defined by rapid and large-scale spread, resulting in high disease prevalence and disease-induced mortality [74]. During the early phase of an SCTLD outbreak, the maximum amount of Symbiodiniaceae diversity should be present on a reef because the most susceptible host and symbiont genotypes have yet to experience widespread mortality. Variations in disease susceptibility are therefore likely most readily detectable during the epidemic phase. As the time from first disease incidence increases, and as the disease transitions from epidemic to endemic, host species and genotypic diversity, as well as Symbiodiniaceae lineage richness, will likely be reduced via mortality of more susceptible lineages or reduced coral abundance [75]. Thus, when a disease has been present on a reef for longer periods of time (i.e. disease has become endemic), fewer differences in Symbiodiniaceae composition across tissue health states may remain. This may be the case for a recent study that documented consistent *Durusdinium* dominance despite reduced SCTLD transmission in *O. faveolata* [76].

The data produced in this study could be used in predictive modeling of coral SCTLD susceptibility. For example, we predict that *C. natans* genets inoculated with *Cladocopium* C3fe/C3-C3fg-C3b-C3ag-C21-C3lr will exhibit increased severity of SCTLD infection (e.g. increased lesion progression rate, increased mortality) or increased prevalence of SCTLD compared to those inoculated with a putatively less susceptible *Durusdinium* lineage (D1/D4/D2-D4c-D1c). In a culture-based study, we predict that the putatively more susceptible Symbiodiniaceae lineages identified here would exhibit earlier and more extreme disease signs (e.g. reduced photosynthetic performance), relative to putatively more resistant lineages. Identifying Symbiodiniaceae lineage-level SCTLD susceptibility is a crucial step in understanding SCTLD etiology and managing ongoing/future disease outbreaks.

These future studies will further test the extent to which associations between Symbiodiniaceae ITS-2 type profiles and SCTLD health states constitute causal relationships of symbiont (and, by extension, holobiont) susceptibility, or whether alternative scenarios are more parsimonious. For example, coral-associated microbial communities can both structure and be structured by host physiology and immune capacity. While host genotype influences the establishment and maintenance of Symbiodiniaceae composition, stressed/diseased hosts may exhibit less control over their symbiont associations (reviewed by [77]), which can lead to dysbiosis (e.g. [78]) more than symbiont SCTLD susceptibility. To date, one study has shown that Symbiodiniaceae composition may influence SCTLD susceptibility between coral conspecifics more than host genotype [44]; additional explicit tests are needed to further clarify and validate host and Symbiodiniaceae disease susceptibility.

### Towards understanding the role of Symbiodiniaceae in SCTLD

This study is the first to identify Symbiodiniaceae ITS-2 type profiles that are associated with apparently healthy versus SCTLD lesion-adjacent tissues, while accounting for variation between coral species and biogeographic regions. Through differential abundance analyses, we identify Symbiodiniaceae lineages enriched or relatively more abundant in certain tissue health states. These lineages were specific to *O. annularis* and *C. natans* in either Florida or the USVI; we identified one *Cladocopium* lineage in USVI *O. annularis*, one *Symbiodinium* and one *Durusdinium* lineage in Florida *C. natans*, and one *Cladocopium* lineage in USVI *C. natans*. We hypothesize a mechanism wherein SCTLD reduces the relative abundance of more susceptible Symbiodiniaceae lineages (i.e. those enriched in apparently healthy tissues of SCTLD-affected corals) and leaves more resistant lineages (i.e. those enriched in lesion-adjacent tissues on diseased corals). Quantifying the relative susceptibility of Symbiodiniaceae lineages to SCTLD can support further exploration of SCTLD etiology (e.g. viral diversity associated with SCTLD-susceptible versus -resistant Symbiodiniaceae lineages) and inform reef restoration efforts (e.g. Symbiodiniaceae lineages used to inoculate corals prior to outplanting).

## Supplementary Material

Web_Material_ycag228

## Data Availability

The raw ITS-2 sequencing data analyzed in this article are available in the NCBI SRA repository at https://www.ncbi.nlm.nih.gov/bioproject/PRJNA1230223, https://www.ncbi.nlm.nih.gov/bioproject/PRJNA955224, and https://www.ncbi.nlm.nih.gov/bioproject/PRJNA1375785, and can be accessed with the accession numbers PRJNA1230223, PRJNA955224, and PRJNA1375785. The SymPortal data generated in this study and sample-associated BioProject accession numbers are available in the online supplementary material and BCO-DMO project 997 771. The code for all analyses can be found in the GitHub repository https://github.com/ckarrick/Symbiodiniaceae_SCTLD.

## References

[1] Precht WF, Gintert BE, Robbart ML. et al. Unprecedented disease-related coral mortality in Southeastern Florida. *Sci Rep* 2016;6:31374. 10.1038/srep31374 PMC4979204 27506875

[2] Kramer P, Roth L, Lang J. Map of stony coral tissue loss disease outbreak in the Caribbean. *ArcGIS Online* 2026. Accessed 2024. www.agrra.org.

[3] Florida Department of Environmental Protection . Case Definition: Stony Coral Tissue Loss Disease (SCTLD). 2018. FL, USA: Florida Department of Environmental Protection, Tallahassee, https://floridadep.gov/sites/default/files/Copy%20of%20StonyCoralTissueLossDisease_CaseDefinition%20final%2010022018.pdf.

[4] Meiling S, Muller EM, Smith TB. et al. 3D photogrammetry reveals dynamics of stony coral tissue loss disease (SCTLD) lesion progression across a thermal stress event. *Front Mar Sci* 2020;7:597643. 10.3389/fmars.2020.597643

[5] Estrada-Saldívar N, Molina-Hernández A, Pérez-Cervantes E. et al. Reef-scale impacts of the stony coral tissue loss disease outbreak. *Coral Reefs* 2020;39:861–6. 10.1007/s00338-020-01949-z

[6] Estrada-Saldívar N, Quiroga-García BA, Pérez-Cervantes E. et al. Effects of the stony coral tissue loss disease outbreak on coral communities and the benthic composition of Cozumel reefs. *Front Mar Sci* 2021;8:632777. 10.3389/fmars.2021.632777

[7] Walton CJ, Hayes NK, Gilliam DS. Impacts of a regional, multi-year, multi-species coral disease outbreak in Southeast Florida. *Front Mar Sci* 2018;5:323. 10.3389/fmars.2018.00323

[8] Papke E, Carreiro A, Dennison C. et al. Stony coral tissue loss disease: a review of emergence, impacts, etiology, diagnostics, and intervention. *Front Mar Sci* 2024;10:1321271. 10.3389/fmars.2023.1321271

[9] Blackall LL, Wilson B, Van Oppen MJH. Coral—the world’s most diverse symbiotic ecosystem. *Mol Ecol* 2015;24:5330–47. 10.1111/mec.13400 26414414

[10] Arriaga-Piñón ZP, Aguayo-Leyva JE, Álvarez-Filip L. et al. Microbiomes of three coral species in the Mexican Caribbean and their shifts associated with the stony coral tissue loss disease. *PLoS One* 2024;19:e0304925. 10.1371/journal.pone.0304925 PMC11346732 39186575

[11] Becker CC, Brandt M, Miller CA. et al. Microbial bioindicators of stony coral tissue loss disease identified in corals and overlying waters using a rapid field-based sequencing approach. *Environ Microbiol* 2021;24:1166–82. 10.1111/1462-2920.15718 34431191

[12] Becker CC, Weber L, Llopiz JK. et al. Microorganisms uniquely capture and predict stony coral tissue loss disease and hurricane disturbance impacts on US Virgin Island reefs. *Environ Microbiol* 2024;26:e16610. 10.1111/1462-2920.16610 38576217

[13] Clark AS, Williams SD, Maxwell K. et al. Characterization of the microbiome of corals with stony coral tissue loss disease along Florida’s coral reef. *Microorganisms* 2021;9:2181. 10.3390/microorganisms9112181 PMC8623284 34835306

[14] Evans JS, Paul VJ, Kellogg CA. Biofilms as potential reservoirs of stony coral tissue loss disease. *Front Mar Sci* 2022;9:1009407. 10.3389/fmars.2022.1009407

[15] Evans JS, Paul VJ, Ushijima B. et al. Investigating microbial size classes associated with the transmission of stony coral tissue loss disease (SCTLD). *PeerJ* 2023;11:e15836. 10.7717/peerj.15836 PMC10460154 37637172

[16] Huntley N, Brandt ME, Becker CC. et al. Experimental transmission of stony coral tissue loss disease results in differential microbial responses within coral mucus and tissue. *ISME Commun* 2022;2:46. 10.1038/s43705-022-00126-3 PMC9723713 37938315

[17] Meyer JL, Castellanos-Gell J, Aeby GS. et al. Microbial community shifts associated with the ongoing stony coral tissue loss disease outbreak on the Florida reef tract. *Front Microbiol* 2019;10:2244. 10.3389/fmicb.2019.02244 PMC6769089 31608047

[18] Rosales SM, Clark AS, Huebner LK. et al. Rhodobacterales and Rhizobiales are associated with stony coral tissue loss disease and its suspected sources of transmission. *Front Microbiol* 2020;11:681. 10.3389/fmicb.2020.00681 PMC7212369 32425901

[19] Rosales SM, Huebner LK, Clark AS. et al. Bacterial metabolic potential and micro-eukaryotes enriched in stony coral tissue loss disease lesions. *Front Mar Sci* 2022;8:776859. 10.3389/fmars.2021.776859

[20] Rosales SM, Huebner LK, Evans JS. et al. A meta-analysis of the stony coral tissue loss disease microbiome finds key bacteria in unaffected and lesion tissue in diseased colonies. *ISME Commun* 2023;3:19. 10.1038/s43705-023-00220-0 PMC9998881 36894742

[21] Thome PE, Rivera-Ortega J, Rodríguez-Villalobos JC. et al. Local dynamics of a white syndrome outbreak and changes in the microbial community associated with colonies of the scleractinian brain coral Pseudodiploria strigosa. *PeerJ* 2021;9:e10695. 10.7717/peerj.10695 PMC7863780 33604172

[22] Vega Thurber R, Correa AMS, Coy S. et al. Meta-Transcriptomics to Determine if and how Viruses Are Involved in SCTLD Infection Status and/or Disease Susceptibility, Tallahassee, FL, USA: Florida Department of Environmental Protection, https://floridadep.gov/rcp/coral-protection-restoration/documents/meta-transcriptomics-determine-if-and-how-viruses-are-0. (31 August 2023).

[23] Veglia AJ . Detecting and interpreting viral dynamics in marine invertebrate Holobionts. *Diss, Rice University* 2023. https://hdl.handle.net/1911/115214.

[24] Veglia AJ, Beavers K, Van Buren EW. et al. Alphaflexivirus genomes in stony coral tissue loss disease-affected, disease-exposed, and disease-unexposed coral colonies in the U.S. Virgin Islands. *Microbiol Resour Announc* 2022;11:e01199–21. 10.1128/mra.01199-21 PMC8852308 35175123

[25] Vega Thurber RL, Silva D, Croquer A. et al. Coral disease: direct and indirect agents, mechanisms of disease, and innovations for increasing resistance and resilience. *Annu Rev Mar Sci* 2025;17:227–55. 10.1146/annurev-marine-011123-102337 39227183

[26] Beavers KM, Van Buren EW, Rossin AM. et al. Stony coral tissue loss disease induces transcriptional signatures of in situ degradation of dysfunctional Symbiodiniaceae. *Nat Commun* 2023;14:2915. 10.1038/s41467-023-38612-4 PMC10202950 37217477

[27] Landsberg JH, Kiryu Y, Peters EC. et al. Stony coral tissue loss disease in Florida is associated with disruption of host–zooxanthellae physiology. *Front Mar Sci* 2020;7:576013. 10.3389/fmars.2020.576013

[28] Meiling SS, Muller EM, Lasseigne D. et al. Variable species responses to experimental stony coral tissue loss disease (SCTLD) exposure. *Front Mar Sci* 2021;8:670829. 10.3389/fmars.2021.670829

[29] Work TM, Weatherby TM, Landsberg JH. et al. Viral-like particles are associated with endosymbiont pathology in Florida corals affected by stony coral tissue loss disease. *Front Mar Sci* 2021;8:750658. 10.3389/fmars.2021.750658

[30] Howe-Kerr LI, Knochel AM, Meyer MD. et al. Filamentous virus-like particles are present in coral dinoflagellates across genera and ocean basins. *ISME J* 2023;17:2389–402. 10.1038/s41396-023-01526-6 PMC10689786 37907732

[31] Work TM, Miller J, Kelley T. et al. Pathology of lesions in corals from the US Virgin Islands after emergence of stony coral tissue loss disease. *Coral Reefs* 2025;44:179–92. 10.1007/s00338-024-02595-5

[32] Beavers KM, Gutierrez-Andrade D, Van Buren EW. et al. Machine learning reveals distinct gene expression signatures across tissue states in stony coral tissue loss disease. *R Soc Open Sci* 2025;12:241993. 10.1098/rsos.241993 PMC12289216 40708668

[33] Studivan MS, Eckert RJ, Shilling E. et al. Stony coral tissue loss disease intervention with amoxicillin leads to a reversal of disease-modulated gene expression pathways. *Mol Ecol* 2023;32:5394–413. 10.1111/mec.17110 37646698

[34] Van Buren EW, Beavers KM, Cornelio MN. et al. Leveraging machine learning to classify and characterize gene expression patterns in two coral diseases. *Discov Appl Sci* 2025;7:1003. 10.1007/s42452-025-07382-7

[35] Deutsch JM, Jaiyesimi OA, Pitts KA. et al. Metabolomics of healthy and stony coral tissue loss disease affected Montastraea cavernosa corals. *Front Mar Sci* 2021;8:714778. 10.3389/fmars.2021.714778

[36] Baker AC . Flexibility and specificity in coral-algal symbiosis: diversity, ecology, and biogeography of Symbiodinium. *Annu Rev Ecol Evol Syst* 2003;34:661–89. 10.1146/annurev.ecolsys.34.011802.132417

[37] Correa AMS, Brandt ME, Smith TB. et al. Symbiodinium associations with diseased and healthy scleractinian corals. *Coral Reefs* 2009;28:437–48. 10.1007/s00338-008-0464-6

[38] Finney JC, Pettay DT, Sampayo EM. et al. The relative significance of host–habitat, depth, and geography on the ecology, endemism, and speciation of coral endosymbionts in the genus Symbiodinium. *Microb Ecol* 2010;60:250–63. 10.1007/s00248-010-9681-y 20502891

[39] LaJeunesse TC, Bhagooli R, Hidaka M. et al. Closely related Symbiodinium spp. differ in relative dominance in coral reef host communities across environmental, latitudinal and biogeographic gradients. *Mar Ecol Prog Ser* 2004;284:147–61. 10.3354/meps284147

[40] Dennison CE , Karp RF, Weiler BA. et al. The Role of Algal Symbionts (Genus Breviolum) in the Susceptibility of Corals to Stony Coral Tissue Loss Disease in South Florida, Tallahassee, FL, USA: Florida Department of Environmental Protection, 2021. https://floridadep.gov/sites/default/files/Baker_UM_Algal%20Symbionts%20Final%20Report_06.2021_508.pdf.

[41] Karp R, Dennison C, Kron K. et al. The Use of Algal Symbiont Cultures (Family Symbiodiniaceae) as Model Systems to Study Stony Coral Tissue Loss Disease: Use of Fractionated Disease Isolates to Help with Pathogen Identification, Tallahassee, FL, USA: Florida Department of Environmental Protection, 2023. https://floridadep.gov/sites/default/files/2023_Final%20Report%20C0A70C_1.pdf.

[42] Eaton KR, Banister RB, Arick LN. et al. Genotype and symbiont composition rather than environment influence susceptibility to stony coral tissue loss disease in coral restoration broodstock. *Sci Rep* 2025;15:44400. 10.1038/s41598-025-29292-9 PMC12727833 41326536

[43] Titus K, O’Connell L, Matthee K. et al. The influence of Foureye butterflyfish (Chaetodon capistratus) and Symbiodiniaceae on the transmission of stony coral tissue loss disease. *Front Mar Sci* 2022;9:800423. 10.3389/fmars.2022.800423

[44] Klein AM, Sturm AB, Eckert RJ. et al. Algal symbiont genera but not coral host genotypes correlate to stony coral tissue loss disease susceptibility among Orbicella faveolata colonies in South Florida. *Front Mar Sci* 2024;11:1287457. 10.3389/fmars.2024.1287457

[45] Davies SW, Gamache MH, Howe-Kerr LI. et al. Building consensus around the assessment and interpretation of Symbiodiniaceae diversity. *PeerJ* 2023;11:e15023. 10.7717/peerj.15023 PMC10162043 37151292

[46] Brandt ME, Ennis RS, Meiling SS. et al. The emergence and initial impact of stony coral tissue loss disease (SCTLD) in the United States Virgin Islands. *Front Mar Sci* 2021;8:715329. 10.3389/fmars.2021.715329

[47] Rossin AM, Beavers KM, Karrick CE. et al. Runaway coral-algal dysbiosis may be responsible for rapid coral tissue loss. *Sci Rep* 2026;16:6415. 10.1038/s41598-026-35666-4 PMC12909295 41593249

[48] Schneider CA, Rasband WS, Eliceiri KW. NIH image to ImageJ: 25 years of image analysis. *Nat Methods* 2012;9:671–5. 10.1038/nmeth.2089 PMC5554542 22930834

[49] Hume BCC, Ziegler M, Poulain J. et al. An improved primer set and amplification protocol with increased specificity and sensitivity targeting the Symbiodinium ITS2 region. *PeerJ* 2018;6:e4816. 10.7717/peerj.4816 PMC5970565 29844969

[50] Hume BCC, Smith EG, Ziegler M. et al. SymPortal: a novel analytical framework and platform for coral algal symbiont next-generation sequencing *ITS2* profiling. *Mol Ecol Resour* 2019;19:1063–80. 10.1111/1755-0998.13004 PMC6618109 30740899

[51] R Core Team . R: A Language and Environment for Statistical Computing. Vienna, Austria: R Foundation for Statistical Computing, 2024, 2024.

[52] McMurdie PJ, Holmes S. Phyloseq: an R package for reproducible interactive analysis and graphics of microbiome census data. *PLoS One* 2013;8:e61217. 10.1371/journal.pone.0061217 PMC3632530 23630581

[53] Wickham H, Averick M, Bryan J. et al. Welcome to the Tidyverse. *J Open Source Softw* 2019;4:1686. 10.21105/joss.01686

[54] Oksanen J, Simpson GL, Blanchet FG. et al. Vegan: Community Ecology Package, 2025. Vienna, Austria: CRAN.

[55] Kassambara A . rstatix: Pipe-Friendly Framework for Basic Statistical Tests, 2025. Vienna, Austria: CRAN.

[56] Mangiafico SS . rcompanion: Functions to Support Extension Education Program Evaluation. New Brunswick, New Jersey: Rutgers Cooperative Extension, 2026.

[57] Saad OS, Lin X, Ng TY. et al. Genome size, rDNA copy, and qPCR assays for Symbiodiniaceae. *Front Microbiol* 2020;11:847. 10.3389/fmicb.2020.00847 PMC7264167 32528423

[58] Wickham H . ggplot2: Elegant Graphics for Data Analysis. New York, NY, USA: Springer-Verl NY, 2016.

[59] Martinez AP . pairwiseAdonis: Pairwise Multilevel Comparison Using Adonis. R package version 0.4.1, 2017, commit cb190f7668a0c82c0b0853927db239e7b9ec3e83.

[60] de VA, Ripley BD. ggdendro: Create Dendrograms and Tree Diagrams Using ‘ggplot2’, 2024. Vienna, Austria: CRAN.

[61] Lin H, Peddada SD. Analysis of compositions of microbiomes with bias correction. *Nat Commun* 2020;11:3514. 10.1038/s41467-020-17041-7 PMC7360769 32665548

[62] Aeby G, Ushijima B, Bartels E. et al. Changing stony coral tissue loss disease dynamics through time in Montastraea cavernosa. *Front Mar Sci* 2021;8:699075. 10.3389/fmars.2021.699075

[63] Baird A, Cumbo V, Leggat W. et al. Fidelity and flexibility in coral symbioses. *Mar Ecol Prog Ser* 2007;347:307–9. 10.3354/meps07220

[64] Baker AC, Romanski AM. Multiple symbiotic partnerships are common in scleractinian corals, but not in octocorals: comment on Goulet (2006). *Mar Ecol Prog Ser* 2007;335:237–42. 10.3354/meps335237

[65] Silverstein RN, Correa AMS, Baker AC. Specificity is rarely absolute in coral–algal symbiosis: implications for coral response to climate change. *Proc R Soc B Biol Sci* 2012;279:2609–18. 10.1098/rspb.2012.0055 PMC3350700 22367985

[66] LaJeunesse T . Diversity and community structure of symbiotic dinoflagellates from Caribbean coral reefs. *Mar Biol* 2002;141:387–400. 10.1007/s00227-002-0829-2

[67] Rowan R, Knowlton N, Baker A. et al. Landscape ecology of algal symbionts creates variation in episodes of coral bleaching. *Nature* 1997;388:265–9. 10.1038/40843 9230434

[68] Rowan R, Knowlton N. Intraspecific diversity and ecological zonation in coral-algal symbiosis. *Proc Natl Acad Sci* 1995;92:2850–3. 10.1073/pnas.92.7.2850 PMC42316 7708736

[69] Toller WW, Rowan R, Knowlton N. Repopulation of zooxanthellae in the Caribbean corals Montastraea annularis and M. faveolata following experimental and disease-associated bleaching. *Biol Bull* 2001;201:360–73. 10.2307/1543614 11751248

[70] Baker AC, Starger CJ, McClanahan TR. et al. Corals’ adaptive response to climate change. *Nature* 2004;430:741–1. 10.1038/430741a 15306799

[71] Buddemeier RW, Fautin DG. Coral bleaching as an adaptive mechanism. *BioScience* 1993;43:320–6. 10.2307/1312064

[72] Claar DC, Starko S, Tietjen KL. et al. Dynamic symbioses reveal pathways to coral survival through prolonged heatwaves. *Nat Commun* 2020;11:6097. 10.1038/s41467-020-19169-y PMC7723047 33293528

[73] Correa AMS, Baker AC. Disaster taxa in microbially mediated metazoans: how endosymbionts and environmental catastrophes influence the adaptive capacity of reef corals. *Glob Change Biol* 2011;17:68–75. 10.1111/j.1365-2486.2010.02242.x

[74] Muller EM, Sartor C, Alcaraz NI. et al. Spatial epidemiology of the stony-coral-tissue-loss disease in Florida. *Front Mar Sci* 2020;7:163. 10.3389/fmars.2020.00163

[75] Cunning R, Lenz EA, Edmunds PJ. Measuring multi-year changes in the Symbiodiniaceae algae in Caribbean corals on coral-depleted reefs. *PeerJ* 2024;12:e17358. 10.7717/peerj.17358 PMC11141555 38827291

[76] Palacio-Castro AM, Soderberg N, Zagon Z. et al. Elevated temperature decreases stony coral tissue loss disease transmission, with little effect of nutrients. *Sci Rep* 2025;15:22261. 10.1038/s41598-025-06322-0 PMC12214535 40596010

[77] Mansfield KM, Gilmore TD. Innate immunity and cnidarian-Symbiodiniaceae mutualism. *Dev Comp Immunol* 2019;90:199–209. 10.1016/j.dci.2018.09.020 30268783

[78] Howe-Kerr LI, Bachelot B, Wright RM. et al. Symbiont community diversity is more variable in corals that respond poorly to stress. *Glob Change Biol* 2020;26:2220–34. 10.1111/gcb.14999 32048447

