# Supplementary material for: Key coral symbiont lineages (family Symbiodiniaceae) are differentially associated with stony coral tissue loss disease
Source: ISME Commun. 2026 Aug 8;6(1):ycag228. doi: 10.1093/ismeco/ycag228 (PMC13590891; doi:10.1093/ismeco/ycag228)
Supplement: Supplementary file 1 [file web_material_ycag228.zip › Supp_Figs_Tables.docx]

Supplementary Material for:

Key coral symbiont lineages (Family Symbiodiniaceae) are differentially associated with stony coral tissue loss disease (SCTLD)

Carly E. Karrick, Alex J. Veglia, Sonora Meiling, Kerry Maxwell, Lindsay K. Huebner, Daniel M. Holstein, Laura Mydlarz, Marilyn Brandt, Amy Apprill, Erinn M. Muller, Adrienne M.S. Correa

**Figure S1:** Flowchart of filtering steps and analyses conducted on (A) the full dataset (*in situ* and experimental samples) and (B) samples from the transmission experiment. Box colors indicate the analyses performed on a given data subset; dashed boxes indicate samples that were removed from downstream analyses.

**Figure S2:** Rarefaction curve of the sequencing depth (number of reads) versus the number of Symbiodiniaceae internal transcribed spacer-2 (ITS-2) type profiles detected from a sample. A sequencing depth of 1,000 reads was used as a cutoff for inclusion in downstream analyses.

**Figure S3:** Boxplots of the sequencing depth (number of reads) for the Symbiodiniaceae internal transcribed spacer-2 (ITS-2) region in each coral species. Letters above each plot correspond with compact letter displays of pairwise Dunn’s tests with Benjamini-Hochberg corrections. Host species are abbreviated as: PAST: *Porites astreoides*; MCAV: *Montastraea cavernosa*; OANN: *Orbicella annularis*; OFAV: *Orbicella faveolata*; SSID: *Siderastrea siderea*; CNAT: *Colpophyllia natans*; DLAB: *Diploria labyrinthiformis*; MMEA: *Meandrina meandrites*; PSTR: *Pseudodiploria strigosa*.

**Figure S4:** Internal transcribed spacer-2 (ITS-2) copy-corrected relative abundance of Symbiodiniaceae ITS-2 type profiles within apparently healthy *Porites astreoides* collected *in situ* (A) and *P. astreoides* exposed to an *ex situ* stony coral tissue loss disease (SCTLD) transmission experiment (B). Per Coral ID, experimental samples are ordered from initial timepoint (T1), to final timepoint after exposure to a healthy fragment (HH_Tf), to final timepoint after exposure to a SCTLD-affected fragment (HD/DD_Tf). Bars are outlined by Symbiodiniaceae genera and filled by ITS-2 type profiles. Samples are faceted by coral ID (i.e., genotype). All samples were collected from the U.S. Virgin Islands, indicated by black x-axis text. HH: apparently healthy tissue on an apparently healthy coral; HD: apparently healthy tissue on a SCTLD-affected coral; DD: SCTLD lesion-adjacent tissue; EAH: apparently healthy tissue from an apparently healthy coral exposed to a diseased coral in the transmission experiment; PAST: *Porites astreoides*.

**Figure S5:** Internal transcribed spacer-2 (ITS-2) copy-corrected relative abundance of Symbiodiniaceae ITS-2 type profiles within apparently healthy *Montastraea cavernosa* collected *in situ* (A), stony coral tissue loss disease (SCTLD)-affected *M. cavernosa* collected *in situ* (B), and *M. cavernosa* exposed to an *ex situ* SCTLD transmission experiment (C). Per Coral ID, experimental samples are ordered from initial timepoint (T1), to final timepoint after exposure to a healthy fragment (HH_Tf), to final timepoint after exposure to a SCTLD-affected fragment (HD/DD_Tf). Bars are outlined by Symbiodiniaceae genera and filled by ITS-2 type profiles. Samples are faceted by coral ID (i.e., genotype). Red x-axis text indicates samples that were collected from Florida, U.S.A. HH: apparently healthy tissue on an apparently healthy coral; HD: apparently healthy tissue on a SCTLD-affected coral; DD: SCTLD lesion-adjacent tissue; EAH: apparently healthy tissue from an apparently healthy coral exposed to a diseased coral in the transmission experiment; MCAV: *Montastraea cavernosa*.

**Figure S6:** Internal transcribed spacer-2 (ITS-2) copy-corrected relative abundance of Symbiodiniaceae ITS-2 type profiles within apparently healthy *Orbicella annularis* collected *in situ* (A), stony coral tissue loss disease (SCTLD)-affected *O. annularis* collected *in situ* (B), and *O. annularis* exposed to an *ex situ* SCTLD transmission experiment (C). Per Coral ID, experimental samples are ordered from initial timepoint (T1), to final timepoint after exposure to a healthy fragment (HH_Tf), to final timepoint after exposure to a SCTLD-affected fragment (HD/DD_Tf). Bars are outlined by Symbiodiniaceae genera and filled by ITS-2 type profiles. Samples are faceted by coral ID (i.e., genotype). All samples were collected from the U.S. Virgin Islands, indicated by black x-axis text. HH: apparently healthy tissue on an apparently healthy coral; HD: apparently healthy tissue on a SCTLD-affected coral; DD: SCTLD lesion-adjacent tissue; EAH: apparently healthy tissue from an apparently healthy coral exposed to a diseased coral in the transmission experiment; OANN: *Orbicella annularis*.

**Figure S7:** Internal transcribed spacer-2 (ITS-2) copy-corrected relative abundance of Symbiodiniaceae ITS-2 type profiles within apparently healthy and stony coral tissue loss disease (SCTLD)-affected *Orbicella faveolata* collected *in situ*. Bars are outlined by Symbiodiniaceae genera and filled by ITS-2 type profiles. Samples are faceted by coral ID (i.e., genotype). All samples were collected in Florida, U.S.A., indicated by red x-axis text. HH: apparently healthy tissue on an apparently healthy coral; HD: apparently healthy tissue on a SCTLD-affected coral; DD: SCTLD lesion-adjacent tissue; OFAV: *Orbicella faveolata*.

**Figure S8:** Internal transcribed spacer-2 (ITS-2) copy-corrected relative abundance of Symbiodiniaceae ITS-2 type profiles within apparently healthy *Siderastrea siderea* collected *in situ* and *S. siderea* exposed to an *ex situ* stony coral tissue loss disease (SCTLD) transmission experiment. Per Coral ID, experimental samples are ordered from initial timepoint (T1), to final timepoint after exposure to a healthy fragment (HH_Tf), to final timepoint after exposure to a SCTLD-affected fragment (HD/DD_Tf). Bars are outlined by Symbiodiniaceae genera and filled by ITS-2 type profiles. Samples are faceted by coral ID (i.e., genotype). Red x-axis text indicates samples collected from Florida, U.S.A. HH: apparently healthy tissue on an apparently healthy coral; HD: apparently healthy tissue on a SCTLD-affected coral; DD: tissue at the lesion line on a SCTLD-affected coral; EAH: apparently healthy tissue from an apparently healthy coral exposed to a diseased coral in the transmission experiment; SSID: *Siderastrea siderea*.

**Figure S9:** Internal transcribed spacer-2 (ITS-2) copy-corrected relative abundance of Symbiodiniaceae ITS-2 type profiles within apparently healthy *Colpophyllia natans* collected *in situ* (A), stony coral tissue loss disease (SCTLD)-affected *C. natans* collected *in situ* (B), and *C. natans* exposed to an *ex situ* SCTLD transmission experiment (C). Per Coral ID, experimental samples are ordered from initial timepoint (T1), to final timepoint after exposure to a healthy fragment (HH_Tf), to final timepoint after exposure to a SCTLD-affected fragment (HD/DD_Tf). Bars are outlined by Symbiodiniaceae genera and filled by ITS-2 type profiles. Samples are faceted by coral ID (i.e., genotype). Red x-axis text indicates samples collected from Florida, U.S.A. HH: apparently healthy tissue on an apparently healthy coral; HD: apparently healthy tissue on a SCTLD-affected coral; DD: SCTLD lesion-adjacent tissue; CNAT: *Colpophyllia natans*.

**Figure S10:** Internal transcribed spacer-2 (ITS-2) copy-corrected relative abundance of Symbiodiniaceae ITS-2 type profiles within *Diploria labyrinthiformis* exposed to an *ex situ* stony coral tissue loss disease (SCTLD) transmission experiment. Bars are outlined by Symbiodiniaceae genera and filled by ITS-2 type profiles. Samples are faceted by coral ID (i.e., genotype). Per Coral ID, samples are ordered from initial (T1) to final (Tf) timepoint. All samples were collected from a transmission experiment in the U.S. Virgin Islands, indicated by black x-axis text. HH: apparently healthy tissue on an apparently healthy coral; HD: apparently healthy tissue on a SCTLD-affected coral; DD: SCTLD lesion-adjacent tissue; DLAB: *Diploria labyrinthiformis*.

**Figure S11:** Internal transcribed spacer-2 (ITS-2) copy-corrected relative abundance of Symbiodiniaceae ITS-2 type profiles within apparently healthy and stony coral tissue loss disease (SCTLD)-affected *Meandrina meandrites* collected *in situ*. Bars are outlined by Symbiodiniaceae genera and filled by ITS-2 type profiles. Samples are faceted by coral ID (i.e., genotype). Red x-axis text indicates samples collected from Florida, U.S.A. HH: apparently healthy tissue on an apparently healthy coral; HD: apparently healthy tissue on a SCTLD-affected coral; DD: SCTLD lesion-adjacent tissue; MMEA: *Meandrina meandrites*.

**Figure S12:** Internal transcribed spacer-2 (ITS-2) copy-corrected relative abundance of Symbiodiniaceae ITS-2 type profiles within apparently healthy *Pseudodiploria strigosa* collected *in situ* (A), stony coral tissue loss disease (SCTLD)-affected *P. strigosa* collected *in situ* (B), and *P. strigosa* exposed to an *ex situ* SCTLD transmission experiment (C). Per Coral ID, experimental samples are ordered from initial timepoint (T1), to final timepoint after exposure to a healthy fragment (HH_Tf), to final timepoint after exposure to a SCTLD-affected fragment (HD/DD_Tf). Bars are outlined by Symbiodiniaceae genera and filled by ITS-2 type profiles. Samples are faceted by coral ID (i.e., genotype). Red x-axis text indicates samples collected from Florida, U.S.A. HH: apparently healthy tissue on an apparently healthy coral; HD: apparently healthy tissue on a SCTLD-affected coral; DD: SCTLD lesion-adjacent tissue; EAH: apparently healthy tissue from an apparently healthy coral exposed to a diseased coral in the transmission experiment; PSTR: *Pseudodiploria strigosa*.

**Table S1: Internal transcribed spacer-2 (ITS-2) type profiles identified by SymPortal**

| **Symbiodiniaceae genus** | **# ITS-2 type profiles including initial experimental samples (# excluding these samples)** | **ITS-2 type profiles** |
| --- | --- | --- |
| *Symbiodinium* | 11 (11) | A13 |
|  |  | A4/A4a/A4bz |
|  |  | A4/A4a-A4ah |
|  |  | A4-A4a-A4ef |
|  |  | A4/A4ea-A4a |
|  |  | A4 |
|  |  | A4-A4a-A4.3-A4s-A4e |
|  |  | A13i |
|  |  | A4/A4a |
|  |  | A4a/A4-A4.3 |
|  |  | A1es/A4 |
| *Breviolum* | 53 (52) | B19-B5af-B19e-B19d-B19g-B19c-B40a-B19f-B5ag |
|  |  | B1-B1x-B1t-B14b-B1af-B1ae-B1ag-B1bh-B1be |
|  |  | B1-B14b-B14d-B1x-B1co-B1ai-B14i-B1j-B1az |
|  |  | B6/B6d/B6e-B6b-B19i-B19af-B6f-B6a-B18g-B19ag-B6c-B6g |
|  |  | B1-B14b-B1x-B14d-B1co-B1ai-B1be-B14i |
|  |  | B1-B14d-B1co-B1az-B14i-B1x-B1ai-B5dy |
|  |  | B1/B14d-B1co-B1az-B1x-B14i-B1ai-B14b |
|  |  | B1-B1z-B1bi-B1aa-B1ad-B1ac |
|  |  | B1-B1cv-B10-B1do |
|  |  | B1-B1da-B1cx-B1gu-B1fk-B1cw-B1gx-B1gw-B1gv |
|  |  | B5de/B1 |
|  |  | B1-B1z-B1aa-B1ac-B1ab-B1ad |
|  |  | B1-B1as-B1ci |
|  |  | B6-B19af-B6d-B6b-B19i-B6e-B6a-B6f-B6c-B19ag |
|  |  | B6-B6d-B19i-B6a-B6e-B6c-B19af-B6f-B19bg-B6b |
|  |  | B1-B1gr |
|  |  | B1-B1ae |
|  |  | B19i/B6g-B6-B6a-B6i-B6b-B19af-B19as-B6h-B6c |
|  |  | B1-B1z-B1bi-B1g-B1ac-B1aa |
|  |  | B6b/B6d/B1-B6-B19af-B19i-B14d-B6a-B14i |
|  |  | B19/B1-B5af-B19e-B19d-B19g-B19c |
|  |  | B1-B5dx-B1gs-B1gt |
|  |  | B6/B19i/B6e-B6b-B19af-B6a-B19as-B6g |
|  |  | B1bl/B1-B1x-B1t |
|  |  | B1 |
|  |  | B1/B19i-B6g-B6-B6a-B6i-B6h-B19as-B6b-B19af |
|  |  | B1/B19 |
|  |  | B19bh |
|  |  | B1/B5 |
|  |  | B1-B1z-B1ad-B1bi-B1aa |
|  |  | B1-B1ag-B1ae-B1x-B1t-B14b-B1af-B1az |
|  |  | B1-B1as-B1cg-B1e |
|  |  | B19-B19ak |
|  |  | B19i/B6-B6f-B19af-B6a-B6d-B6e-B6c-B6b-B18g |
|  |  | B6-B6e-B6a-B6f-B19i-B19ag-B19af-B6d-B6b |
|  |  | B1-B14b-B1x-B1ae-B1af-B1t |
|  |  | B1-B19i-B6-B19af-B6a |
|  |  | B6d-B6b-B6-B19i |
|  |  | B19/B5-B5a |
|  |  | B1-B1ed-B1ci |
|  |  | B5a/B5 |
|  |  | B1-B1bs |
|  |  | B19/B19ai |
|  |  | B5 |
|  |  | B19au |
|  |  | B1-B1g |
|  |  | B1-B1ca |
|  |  | B1-B1s |
|  |  | B1-B1g-B1s-B1bz |
|  |  | B2/B1 |
|  |  | B6-B19af-B19i-B6d-B6a-B6e-B6f-B6b-B6c-B19ag-B18g |
|  |  | B1-B1x-B14d-B14b |
|  |  | B19-B19ax-B1 |
| *Cladocopium* | 32 (28) | C3fe/C3-C3fg-C3b-C3ag-C21-C3lr |
|  |  | C7/C12c/C7d-C12b-C12e-C7c-C7b |
|  |  | C3/C3bb-C3de-C21ae-C3s-C3an-C21-C3dk-C3abv |
|  |  | C3-C3de-C3fc-C3an-C21-C3bb-C21ae-C3b-C3s |
|  |  | C3-C3fc-C21-C3b-C3an-C3fd-C3s-C3bb |
|  |  | C3-C3de-C3bb-C21ae-C3an-C3s-C21-C3dk-C3ji |
|  |  | C54a/C54b-C54q-C3-C54d-C54c |
|  |  | C3-C16a |
|  |  | C7/C12c/C3fe-C3-C7d-C12b-C3fg-C12e-C3b |
|  |  | C12c/C7/C7d-C12b-C12e-C7h-C12f |
|  |  | C7/C12c/C7d-C12b-C12e-C7c |
|  |  | C3 |
|  |  | C3/C21-C3b |
|  |  | C7-C12c-C7d-C12b-C12e |
|  |  | C3-C3c |
|  |  | C7/C12c/C7d-C12b-C12e-C7s-C12i |
|  |  | C3-C21-C3fc-C3an-C3bb-C3b-C3s-C3de |
|  |  | C3/C3bb-C3de-C21ae-C3an-C21-C3s-C3dk |
|  |  | C3-C1-C16a |
|  |  | C12c/C7-C7d-C12b-C12e-C7h |
|  |  | C3-C3b-C3an-C21-C3s |
|  |  | C3-C3ed |
|  |  | C44/C47/C42.2 |
|  |  | C1 |
|  |  | C1/C1b |
|  |  | C3-C3ag-C3b-C21-C3bb |
|  |  | C3-C3lp |
|  |  | C3ag |
|  |  | C3-C40g-C3fc-C3b-C3an-C21-C3fd-C3s |
|  |  | C7/C7d-C12c-C12b |
|  |  | C3fe-C3-C3b-C3ag-C3fg-C21 |
|  |  | C7/C3/C7d-C12c-C12b-C12e |
| *Durusdinium* | 11 (10) | D1-D4-D4c-D1c-D1k-D2 |
|  |  | D1/D4/D2-D4c-D1c |
|  |  | D1-D4-D4c-D2-D1c-D17d |
|  |  | D1-D4-D4c-D1hg-D1c-D2 |
|  |  | D1/D4-D4c-D1c-D2-D17d-D1r-D17c-D17e |
|  |  | D1/D4 |
|  |  | D1/D4-D1c |
|  |  | D1-D4-D4c-D2-D4f |
|  |  | D1-D4-D4c-D2-D1c-D1ip |
|  |  | D1-D4-D4c-D2-D1k-D4i |
|  |  | D1-D4-D4c-D1c-D2-D1k-D17d |
| *Fugacium* | 2 (2) | F3az |
|  |  | F3ba |
| *Gerakladium* | 5 (5) | G3d/G3e-G3c-G3f-G3h-G3g |
|  |  | G3b/G3al-G3ay-G3bs |
|  |  | G3l |
|  |  | G3d-G3c-G3e-G3h-G3f |
|  |  | G3b |

**Table S1:** The internal transcribed spacer-2 (ITS-2) type profiles representing different Symbiodiniaceae genera including and excluding initial experimental samples. Sample sizes of ITS-2 type profiles per Symbiodiniaceae genus are shown for the dataset including initial experimental samples, and the number of ITS-2 type profiles excluding the initial experimental samples are shown in parentheses. ITS-2 type profiles highlighted in gray are not present in the dataset excluding the initial experimental samples.

**Table S2:** **Copy-number corrected (CNC) PERMANOVA results**

| **Dataset** | **Variable** | **Df** | **SumOfSqs** | **R2** | **F** | **p** |
| --- | --- | --- | --- | --- | --- | --- |
| CNC_no_exp_t1 | health | 3 | 1.4241 | 0.21337 | 0.9946 | 0.506 |
| CNC_no_exp_t1 | susceptibility | 2 | 1.6519 | 0.2475 | 1.9735 | 0.008* |
| CNC_no_exp_t1 | moderate_vs_low | 1 | 0.9 | 0.21951 | 2.25 | 0.097 |
| CNC_no_exp_t1 | high_vs_low | 1 | 0.9889 | 0.24654 | 2.6176 | 0.038* |
| CNC_no_exp_t1 | high_vs_moderate | 1 | 0.5889 | 0.1335 | 1.2326 | 0.43 |
| PAST_CNC  _no_exp_t1 | health | 3 | 0.5 | 0.333333 | 0.75 | 1 |
| MCAV_CNC  _no_exp_t1 | region | 1 | 7.4463 | 0.2913 | 27.128 | 0.001* |
| MCAV_FL_CNC  _no_exp_t1 | health | 3 | 0.2988 | 0.03446 | 0.6067 | 0.583 |
| MCAV_USVI_CNC  _no_exp_t1 | health | 3 | 1.1898 | 0.12587 | 1.2971 | 0.252 |
| OANN_CNC  _no_exp_t1 | health | 3 | 1.5647 | 0.10736 | 1.5635 | 0.1 |
| SSID_CNC  _no_exp_t1 | health | 3 | 1.3348 | 0.18767 | 1.0781 | 0.373 |
| CNAT_CNC  _no_exp_t1 | region | 1 | 7.067 | 0.19524 | 20.864 | 0.001* |
| CNAT_FL_CNC  _no_exp_t1 | health | 2 | 0.4483 | 0.01868 | 0.5329 | 0.939 |
| CNAT_USVI_CNC  _no_exp_t1 | health | 2 | 0.6746 | 0.09121 | 1.3548 | 0.241 |
| DLAB_CNC  _no_exp_t1 | health | 1 | 0.4044 | 0.10842 | 1.0945 | 0.363 |
| MMEA_CNC  _no_exp_t1 | region | 1 | 2.3561 | 0.38596 | 9.4284 | 0.001* |
| MMEA_FL_CNC  _no_exp_t1 | health | 2 | 0.87346 | 0.31305 | 1.3671 | 0.25 |
| MMEA_USVI_CNC  _no_exp_t1 | health | 2 | 0.88426 | 0.92271 | 29.844 | 0.056 |
| PSTR_CNC  _no_exp_t1 | region | 1 | 5.8220 | 0.27570 | 19.793 | 0.001* |
| PSTR_FL_CNC  _no_exp_t1 | health | 2 | 0.6054 | 0.02931 | 0.7852 | 0.614 |
| PSTR_USVI_CNC  _no_exp_t1 | health | 2 | 0.06816 | 0.01360 | 0.1655 | 0.965 |

**Table S2:** Copy-number corrected (CNC) PERMANOVA results excluding initial experimental (exp_t1) samples. Pairwise results are shown underneath the global result if the global PERMANOVA for the variables “health” or “susceptibility” yielded a p < 0.05 (indicated by *). If the global PERMANOVA for the variable “region” within coral species subsets of the data yielded a p < 0.05, PERMANOVAs were conducted within regional subsets of the coral species datasets.

**Table S3: Results of Kruskal-Wallis tests**

| **Coral species** | **χ^2^** | **df** | **p value** |
| --- | --- | --- | --- |
| *Diploria labyrinthiformis* | 0.430555555555554 | 2 | 0.806317432002076 |
| *Colpophyllia natans* | 3.88888888888889 | 2 | 0.143066682754408 |
| *Montastraea cavernosa* | 0 | 1 | 1 |
| *Siderastrea siderea* | 1.4 | 2 | 0.49658530379141 |
| *Porites astreoides* | 1.8 | 2 | 0.406569659740599 |
| *Pseudodiploria strigosa* | 0.600000000000001 | 2 | 0.740818220681717 |

**Table S3:** Results of Kruskal-Wallis tests comparing mean lesion progression rate across samples dominated by distinct internal transcribed spacer-2 (ITS-2) type profiles within coral species in the stony coral tissue loss disease (SCTLD) transmission experiment. *O. annularis* is not shown here because all samples of this species were dominated by the same ITS-2 type profile.
